# Forgone healthcare and financial burden due to out-of-pocket payments in Bangladesh: a multilevel analysis

**DOI:** 10.1186/s13561-021-00348-6

**Published:** 2022-01-10

**Authors:** Md. Mizanur Rahman, Md. Rashedul Islam, Md. Shafiur Rahman, Fahima Hossain, Ashraful Alam, Md. Obaidur Rahman, Jenny Jung, Shamima Akter

**Affiliations:** 1 Hitotsubashi Institute for Advanced Study, Hitotsubashi University, 2-1 Naka Kunitachi, Tokyo, 186-8601 Japan; 2 Department of Global Health Policy, School of International Health, The University of Tokyo, Tokyo, Japan; 3 Research Centre for Child Mental Development, Hamamatsu University School of Medicine, Hamamatsu, Japan; 4 Global Public Health Research Foundation, Dhaka, Bangladesh; 5 Department of Global Health Nursing, Graduate School of Nursing Science, St. Luke’s International University, Tokyo, Japan

**Keywords:** Out-of-pocket health payment, Catastrophic health expenditure, Forgone healthcare, Multilevel analysis, Bangladesh

## Abstract

**Background:**

Ensuring access to health services for all is the main goal of universal health coverage (UHC) plan. Out-of-pocket (OOP) payment still remains the main source of funding for healthcare in Bangladesh. The association between barriers to accessing healthcare and over-reliance on OOP payments has not been explored in Bangladesh using nationally representative household survey data. This study is a novel attempt to examine the burden of OOP payment and forgone healthcare in Bangladesh, and further explores the inequalities in catastrophic health expenditures (CHE) and forgone healthcare at the national and sub-national levels.

**Methods:**

This study used data from the most recent nationally representative cross-sectional survey, Bangladesh Household Income and Expenditure Survey, conducted in 2016–17 (*N* = 39,124). In order to identify potential determinants of CHE and forgone healthcare, multilevel Poisson regression was used. Inequalities in CHE and forgone healthcare were measured using the slope index of inequality.

**Results:**

Around 25% of individuals incurred CHE and 14% of the population had forgone healthcare for any reasons. The most common reasons for forgone healthcare were treatment cost (17%), followed by none to accompany or need for permission (5%), and distance to health facility (3%). Multilevel analysis indicated that financial burden and forgone care was higher among households with older populations or chronic illness, and those who utilize either public or private health facilities. Household consumption quintile had a linear negative association with forgone care and positive association with CHE.

**Conclusion:**

This study calls for incorporation of social safety net in health financing system, increase health facility, and gives priority to the disadvantaged population to ensure access to health services for all.

**Supplementary Information:**

The online version contains supplementary material available at 10.1186/s13561-021-00348-6.

## Background

Ensuring access to quality health services for all and protecting population from financial hardship when they receive services is the key health-related target of the Sustainable Development Goals (SDGs) that all member states of the United Nations have agreed to achieve by 2030 [1, 2]. However, in 2015, approximately 808 million people globally experienced catastrophic health expenditure (CHE) [3], and more than 97 million people were pushed below the poverty line due to out-of-pocket (OOP) payment for hea﻿lt﻿﻿h ﻿care [4]. Overreliance on OOP payments, poor functioning of health insurance plans, and different socio-demographic and cultural factors were found to hinder people’s access to health care in times of need [5–9]. Identifying dimensions of barriers to access to health care is therefore crucial for helping policy makers to address the target population and ensure access for all citizens.

In low- and middle-income countries (LMICs), substantial improvements in access to health services have been achieved in the last few decades. However, the inequalities in the healthcare of LMICs persistently deprive their most disadvantaged and socially excluded populations of the benefits of access to health service and desirable health outcomes. To achieve access and quality of care for all populations, Tanahashi [8], Levesque [9], and other experts [5, 10] developed a remarkable health coverage framework based on three dimensions: availability, accessibility, and acceptability. Availability mainly includes human power, facilities, and drugs; accessibility considers distance, and cost related to user fee and transportation; and acceptability includes wide range of socio-demographic, and other demand side characteristics. These three dimensions are mainly responsible for directing the coverage, access, quality, delayed or forgone health care in any countries [5, 8–10].

Like many LMICs, the health financing system in Bangladesh is underfunded, lacking of proper health insurance, and heavily relies on OOP payment [11]. The OOP constitutes 67% of the total health expenditure in Bangladesh [12], and wide pro-rich inequalities with low coverage is observed in most of the health interventions [13, 14]. As a result of higher dependency on OOP payments, around 16% households incurred financial catastrophe in 2011, 5% non-poor households became poor, and 7% of Bangladeshi households experienced distress financing such as borrow money, loan or sell assets to cope up with unpredictable OOP payment [13, 14]. Although, studies on the levels and determinants of financial hardship are not new in Bangladesh [13], little is known to identify populations at high risk of foregoing healthcare in context of Tanahashi barrier dimension such as availability, accessibility and acceptability reasons [8]. To address these questions, we used most recent nationally representative household level survey data to (a) explore the burden of OOP payments for healthcare, and (b) to estimate prevalence and determinants of foregone health care due to availability, accessibility and acceptability reasons using multilevel modeling.

## Methods

### Data sources

Household Income and Expenditure Survey (HIES) conducted during 2016–17 in Bangladesh was used in this study. The HIES is a two-stage stratified sample design and the survey covered eight administrative regions of the country namely Barisal, Chittagong, Dhaka, Khulna, Mymensingh, Rajshahi, Rangpur, and Sylhet. In the first stage of sampling, 2304 primary sampling units (PSU) were selected from the list of the 2011 Housing and Population Census enumerations area (CEA). In the second stage, a fixed number of 20 households were selected from each of the selected PSU making the final sample size to be 46,080 households. In our study, we included participants if they reported any illness or injuries in the 12 months or 30 days recall periods. Based on this criteria, 39,124 household members were included in this study for analysis. The key characteristic of this survey included an integrated household questionnaire which covered household roster, food consumption expenditure, non-food expenditure, housing, assets, education, durable goods, health, individual level care-seeking behavior, individual level healthcare costs, and sources of financing for healthcare.

### Burden of OOP payment variables

OOP payment was defined as the total amount of money paid by the household while receiving the health care services. These included consultation fees, diagnostic fees, hospital bills, transportation, and expenditure on medications. The variable “financial catastrophe” was defined as the percentage of individual OOP expenses on household consumption expenditure, non-food expenditure, or household capacity to pay in the past year. The determination of the thresholds in analyzing catastrophic expenditure varies. The most common thresholds are either 10% or 15% of total household consumption expenditure [15–17], 25% or 40% for non-food expenditure [6, 16, 18], or 40% household capacity to pay [19]. To compare the findings of other studies, the incidence of CHE was presented based on 40% threshold of non-food expenditure.

### Foregone healthcare determinants

In the HIES, every individual member was asked whether he/she sought any type of medical treatment for their health problems in the last 12 months (“yes”/“no”)? Respondents, who replied “no”, were then asked the main reason for not seeking medical care. Possible answers were: 1) problem was not serious; 2) treatment cost is too much; 3) distance is too long; 4) afraid of discovering serious illness; 5) there was none to accompany; 6) decision maker does not think I should go; 7) don’t know where to go; 8) others reasons. To assess the forgone care due to expense, we constructed binary (“yes”/ “no”) variables and determined whether or not the participants avoided healthcare for its expensive costs.

### Predictor variables

Predictor variables were selected by following Andersen’s Behavior model and other literatures [7, 20–22]. The framework suggests that healthcare utilization is influenced by three main factors: predisposing, enabling, and need. The predisposing component – demographic, social structure, and attitude and beliefs about medical care – are factors that are commonly associated with how an individual will utilize health care services prior to the onset of disease or illness. The enabling component is the financial capacity and resource availability. The need component is based on the immediate reason for why the patient sought health care. Because this study did not evaluate utilization of health services, predictor variables related to the predisposing and enabling factors were included in the analysis. Furthermore, we followed previous studies conducted in LMICs for the selection and categorization of predictor variables. Evidence suggested that disadvantaged households [23–27]. household age structure (≤ 5 year children, ≥ 60 years elderly) [25–28], urban location [25], female household head [28], presence of chronic illness [25–30] and increased illness episodes in children [25, 28] were positively associated with CHE and forgone healthcare. However, households with educated household head were less likely to have CHE and forgone healthcare [25–27, 29]. Administratively, Bangladesh is divided into eight regions- Barisal, Chittagong, Dhaka (Capital), Khulna, Mymensing, Rajshai, Rangpur, and Shylhet. These administrative regions are not homogenously developed. For example, the Rangpur is deprived than other regions. From a policy perspective, evidence of the regional distribution of CHE and forgone healthcare could be of importance to Bangladesh. In this study, we were interested to see the variation of CHE and forgone healthcare by household demography, illness and injury, regional and geographical location, and economic condition such as age of population (0–4, 5–9, 10–14, 15–19, 20–24, 25–64, ≥65 years), gender (male, female), religion (Muslim, Non-Muslim), marital status (never married, currently married, widowed/divorced/separated), presence of chronic illness (yes, no), income earner (yes, no), inpatient care (none, public hospital, private hospital/clinics), outpatient care (none, public providers, private providers, self-medication/pharmacy/traditional healer), household consumption quintile (poorest, poorer, average, richer, richest), and place of residence (rural, urban).

### Statistical analysis

Mean and percentage with 95% confidence interval were used to present study characteristics, CHE, and forgone healthcare at the national and regional level. OOP health expenditure was characterized by excess zeroes corresponding to individuals who reported no health expenditure in the past 12 months. OOP health expenditure also relied on participation in seeking care. Since ordinary regression models are inappropriate to control for two stochastic approaches (participation and level of expenses), we therefore preferred the Heckman selection model over standard linear regression [2, 31]. The Heckman model includes a two-stage procedure: a selection equation including the probability of attending a health service in the first stage and a prediction equation for the model’s outcome variable-level of OOP health expenditure at the second stage. In our study, Heckman selection model was further used to identify the determinants of OOP health payments on medical treatment, conditional upon having sought care from any healthcare provider (yes/no). In the case of rare events, Poisson regression can provide more valid estimates than logistic regression [32, 33]. Because barrier to access healthcare resulting from high OOP payments can be a rare event, in our study three-level Poisson regression models with random intercept at the households and community levels were used to investigate the association between individual-, household-, and community-level characteristics and the selected outcome variables, especially CHE and forgone healthcare. Adjusted relative risk (RR) with their 95% confidence interval (CI) were reported from multilevel Poisson regression model.

The inequalities in CHE and forgone healthcare were measured using regression-based slope index of inequality (SII). The values of SII were expressed in percentage points. Positive value of SII indicates that the rich households have lower forgone healthcare or incur lower financial catastrophe than the poor households. Conversely, the negative value indicates that the poor households have higher forgone healthcare or incur higher financial catastrophe than the rich households. An SII value of zero indicates no inequality. Data management and statistical analysis was conducted using Stata/SE version 16.1 (StataCorp LP, College Station, TX) and R version 4.2.1.

## Results

### Respondents’ characteristics

The characteristics of the study population are shown in Table 1. Of the 39,124 household members, more than half were females (54%), mean age of participants was around 28 years, and average household size was around 5 members. In the study population, 29% suffered from chronic illness, 4% used inpatient care, and 86% used outpatient care. Among outpatient care users, 12% used public health services, 41% used private health services, and 32% used self-medication/pharmacy/traditional healer. Around two-thirds of the study sample lived in rural areas. On average, total annual OOP health expenditure was BD TK 12362.2 (95% CI, 11724.5–12,999.8).

**Table 1:** Respondent characteristics

Variables	Frequency	Values	95% confidence interval	
			Lower	Upper
**Mean (95% CI)**				
Household size	39,124	4.51	4.45	4.56
Age, years	39,124	27.53	27.12	27.93
OOP payments (BD TK)	39,124	12,362.17	11,724.55	12,999.78
**Percentage (95% CI)**				
Gender				
Female	21,248	54.36	53.69	55.04
Male	17,875	45.64	44.96	46.31
Religion				
Muslim	34,758	90.10	88.29	91.65
Non-Muslim	4363	9.90	8.35	11.71
Marital status				
Never married	10,934	34.27	33.26	35.30
Currently married	19,930	58.88	57.86	59.88
Widowed^a^	2277	6.85	6.46	7.27
Have chronic illness				
Yes	12,004	29.00	27.85	30.18
No	27,120	71.00	69.82	72.15
**Inpatient care**				
None	37,312	95.56	95.06	96.01
Public hospital/clinic	1099	2.58	2.27	2.94
Private providers	713	1.86	1.63	2.12
**Outpatient care**				
None	4631	14.08	12.30	16.08
Public hospital/clinic	5214	12.47	11.67	13.31
Private hospital/clinic	16,396	41.34	39.39	43.33
Self-medication^b^	12,883	32.11	30.63	33.62
Income earner				
No	25,490	72.37	71.57	73.16
Yes	9940	27.63	26.84	28.43
Place of residence				
rural	27,712	74.10	71.54	76.51
urban	11,412	25.90	23.49	28.46

^a^Widowed/Divorced/Separated

^b^Self-medication/traditional healer

*BD TK* Bangladesh currency Taka

### Determinants of burden of healthcare expenditure

The determinants of CHE results is presented in Table 2. Overall, around 25% of population incurred CHE at 40% threshold of non-food consumption expenditure. The CHE for other definitions and thresholds such as 10% or 15% of total consumption, 25% of non-food, and 40% of household capacity to pay are presented in the supplemental appendix (Table S1). Almost similar incidence of CHE was found for total consumption and non-food consumption, but higher from household capacity to pay (Table S1). The highest incidence of CHE was found among individuals who utilized inpatient care (public hospital (56%) and private hospital/clinic (70%)), followed by individuals having chronic illness (33%), aged 65 years or more (34%), and belonging to wealthy socioeconomic condition (33%). Multilevel analysis indicated that the risk of CHE was significantly high for participants who were widowed/separated, chronically ill, found to utilize public or private health services, and members of households in the richest quintile. Other determinants of CHE were household size and place of residence. Regarding the level of OOP payment, individuals reporting chronic illness, household size, inpatient and outpatient care-seeking behavior, household consumption quintile, and place of residence significantly affected the level of individual OOP healthcare spending (supplemental appendix, Table S2).

**Table 2:** Multilevel Poisson regression model of risk of catastrophic healthcare expenditure, Bangladesh, 2017 (*N* = 39,124)

Variable	Frequency of catastrophic expenditure	Proportion (95% CI)	RR (95% CI)	*p*-values
**Age, years**				
0–4	1297	19.7 (17.7–21.9)	1.00	
5–9	756	19.5 (16.9–22.4)	0.90 (0.82–0.99)	0.02
10–14	657	22.0 (19.8–24.4)	0.91 (0.84–1.00)	0.05
15–19	572	21.7 (19.3–24.4)	0.89 (0.80–0.98)	0.02
20–24	588	25.9 (23.2–28.7)	0.99 (0.88–1.10)	0.80
25–64	4742	27.1 (25.8–28.4)	0.89 (0.79–1.00)	0.05
≥ 65	944	34.3 (31.6–37.1)	1.05 (0.92–1.19)	0.47
**Gender**				
Male	4326	24.1 (22.7–25.7)	1.00	
Female	5230	25.0 (23.6–26.3)	1.02 (0.98–1.06)	0.38
**Religion**				
Muslim	8657	24.9 (23.6–26.4)	1.00	
Non-Muslim	899	21.3 (18.3–24.7)	0.91 (0.82–1.00)	0.06
**Marital status**				
Never married	2358	22.3 (20.2–24.6)	1.00	
Currently married	5535	28.0 (266–29.3)	1.14 (1.04–1.25)	< 0.01
Widowed^a^	643	28.1 (25.5–31.0)	1.14 (1.01–1.27)	0.03
**Has a chronic disease**				
No	5627	21.2 (19.8–22.6)	1.00	
Yes	3929	32.9 (31.1–34.7)	1.19 (1.14–1.24)	< 0.01
**Outpatient care**				
None	543	11.0 (8.4–14.4)	1.00	
Public hospital	1872	37.7 (34.6–40.9)	2.59 (2.21–3.04)	< 0.01
Private hospital/clinic	5210	32.8 (31.1–34.5)	2.37 (2.03–2.77)	< 0.01
Self-medication^b^	1931	14.9 (13.4–16.5)	1.32 (1.13–1.55)	< 0.01
**Inpatient care**				
None	8469	22.9 (21.6–24.1)	1.00	
Public hospital	597	56.1 (50.3–61.6)	1.58 (1.48–1.69)	< 0.01
Private hospital/clinic	490	69.5 (63.4–75.0)	1.78 (1.60–2.00)	< 0.01
**Income earner**				
Yes	2573	25.7 (24.1–27.4)	1.00	
No	6349	25.4 (24.0–26.9)	1.02 (0.97–1.07)	0.49
**Consumption quintile**				
Q1 (poorest)	970	16.0 (14.3–17.9)	1.00	
Q2	1332	18.6 (16.8–20.5)	1.17 (1.05–1.31)	0.01
Q3	1681	20.5 (18.6–22.4)	1.35 (1.21–1.50)	< 0.01
Q4	2276	27.2 (24.4–30.2)	1.70 (1.53–1.9)	< 0.01
Q5 (richest)	3297	32.8 (29.9–35.8)	2.11 (1.90–2.34)	< 0.01
**Household size**	–	–	0.97 (0.96–0.99)	< 0.01
**Place of residence**				
Rural	7207	26.4 (24.9–27.9)	1.00	
Urban	2349	19.4 (17.1–22.0)	0.71 (0.66–0.77)	< 0.01
Total	9556	24.6 (23.3–25.9)		

*RR* relative risk, *CI* confidence interval

^a^Widowed/Divorced/Separated; ^b^Self-medication/pharmacy /traditional healer

### Prevalence and determinants of forgone healthcare

**Fig. 1:**
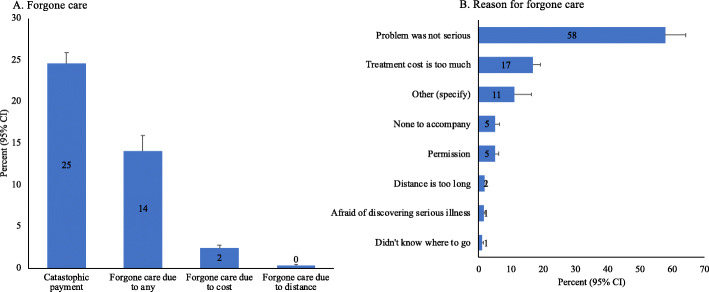
Financial burden, forgone care, and reason for forgone care in Bangladesh, 2017

Prevalence and determinants of forgone healthcare are presented in Fig. 1 and Table 3 respectively. Overall, 14% of the population (4613 out of 39,124 sample) had forgone healthcare for any reasons, including: problem was not serious (58%), cost (17%), none to accompany (5%), distance (3%), and afraid of discovering serious illness (3%). For reasons related to cost of care, around 3% population reported having forgone healthcare because it was unaffordable. Comparatively, a higher proportion of forgone healthcare was evident among elderly populations (4.4%), widowed/divorced/separated (4.7%), reported chronic illness (4.5%), and in lower socio-economic condition (4.5%).

**Table 3:** Multilevel Poisson regression model of risk of forgone healthcare, Bangladesh, 2017 (*N* = 39,124)

Variable	Frequency of forgone healthcare	Proportion (95% CI)	RR (95% CI)	*p*-values
Age, years				
0–4	47	1.0 (0.6–1.5)	1.00	
5–9	54	1.4 (1.1–2.0)	2.03 (1.07–3.87)	0.03
10–14	59	1.8 (1.3–2.5)	2.59 (1.44–4.66)	< 0.01
15–19	58	2.1 (1.5–2.8)	3.15 (1.76–5.65)	< 0.01
20–24	56	2.3 (1.6–3.1)	3.67 (1.93–6.99)	< 0.01
25–64	504	3.0 (3.5–5.6)	3.87 (2.06–7.26)	< 0.01
≥ 65	116	4.4 (3.5–5.6)	4.79 (2.44–9.37)	< 0.01
Gender				
Female	537	2.5 (2.1–2.9)	1.00	
Male	537	2.2 (1.8–2.6)	0.79 (0.65–0.96)	0.02
Religion				
Muslim	802	2.4 (2.0–2.8)	1.00	
Non-Muslim	92	2.2 (1.5–3.2)	0.91 (0.70–1.19)	0.50
Marital status				
Never married	178	1.7 (1.4–2.2)	1.00	
Currently married	545	2.8 (2.4–3.4)	0.81 (0.58–1.14)	0.22
Widowed*	113	4.7 (3.6–6.0)	1.11 (0.74–1.66)	0.61
Has a chronic disease				
No	397	1.5 (1.2–1.7)	1.00	
Yes	498	4.5 (2.8–5.4)	1.73 (1.45–2.07)	< 0.01
Income earner				
No	587	2.4 (2.0–2.8)	1.00	
Yes	278	2.9 (2.4–3.5)	1.09 (0.90–1.32)	0.37
Consumption quintile				
Q1 (poorest)	261	4.5 (3.7–5.5)	1.00	
Q2	146	2.4 (1.8–3.1)	0.47 (0.37–0.60)	< 0.01
Q3	138	1.6 (1.3–2.1)	0.36 (0.28–0.46)	< 0.01
Q4	148	1.8 (1.4–2.5)	0.25 (0.20–0.33)	< 0.01
Q5 (richest)	202	2.2 (1.5–3.2)	0.18 (0.14–0.24)	< 0.01
Household size	–	–	1.06 (1.01–1.11)	0.02
Place of residence				
Rural	680	2.6 (2.2–3.0)	1.00	
Urban	215	1.7 (1.2–2.5)	0.98 (0.76–1.27)	0.90
**Total**	**895**	**2.4 (2.0–2.7)**		

*RR* relative risk, *CI* confidence interval

*Widowed/Divorced/Separated

^a^*p* < 0.01; ^b^*p* < 0.05; ^c^*p* > 0.6

Multilevel regression model revealed that participants’ age, gender, presence of chronic illness, and household consumption quintile were predicted to have significant impacts on foregone healthcare. However, predictors such as religion, marital status, income earner and place of residence were not found to be significant. Forgone healthcare was positively associated with participants’ age and negatively with household consumption quintile. As compared to under five children, the risk of forgone healthcare was significantly higher among all age groups. The highest risk of forgone healthcare was found in populations aged 65 years or over (RR, 4.79; 95% CI, 2.44–9.37) than under five children. The RR representing forgone healthcare among people with chronic illness was 1.73 times higher (95% CI, 1.45–2.07) than those without chronic condition. Participants those in the richest quintile had 82% (RR, 0.18; 95% CI, 0.14–0.24) lower chance of forgone healthcare than those in the poorest quintile.

### Inequality in catastrophic payments and forgone healthcare

**Fig. 2:**
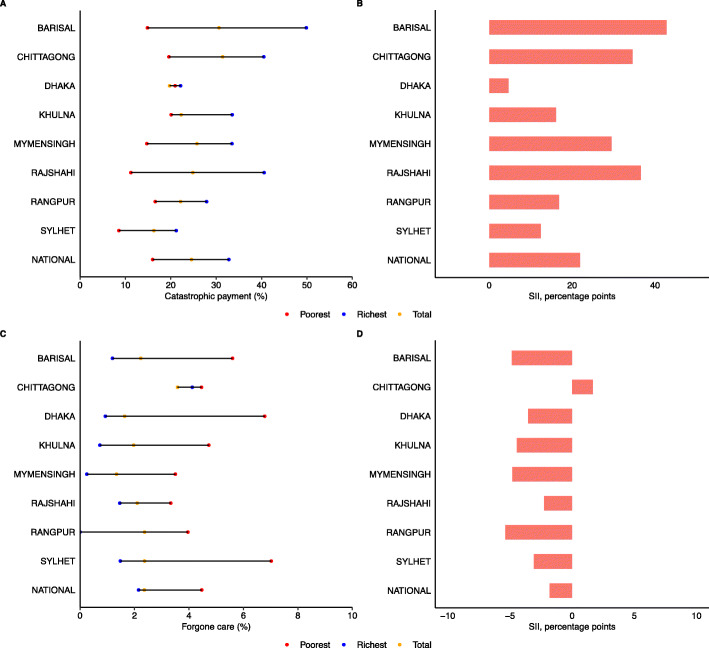
Quintile-specific incidence of catastrophic health payments and forgone healthcare by region in Bangladesh, 2017. Note: A. Household consumption quintile-specific catastrophic health payments (top left); B. Slope index of inequality for catastrophic health payments (top right); D. Household consumption quintile-specific forgone healthcare (bottom left); B. Slope index of inequality for forgone healthcare (bottom right)

Quintile-specific CHE and forgone healthcare by region is presented in Fig. 2. The higher wealth populations were correlated with a higher incidence of financial catastrophe observed at national and all regional levels. A wide disparity was observed both in CHE and forgone healthcare within and between regional levels. The overall incidence of CHE payment was the highest in Chittagong (31.4%) and Barisal (30.6%), and relatively low CHE was observed in Sylhet (16.3%) and Dhaka (19.7%) (Fig. 2A). Wealthier people faced lower financial catastrophe at national and all regional levels than the poorer populations (Fig. 2B). Greater pro-rich inequality in catastrophic payment was evident in Barisal (42.8 percentage points), Rajshahi (36.6 percentage points), and Chittagong (34.6 percentage points), whereas relatively lesser inequality was noticed in Dhaka (4.7 percentage points) and Sylhet (12.4 percentage points). In case of forgone healthcare, the highest proportion of forgone care was observed in Chittagong region (around 4%) and lowest in Mymensingh (around 1%) (Fig. 2C). With exception to Chittagong, pro-poor inequalities were observed in all other regions (Fig. 2D).

## Discussion

The main focus of this study was to assess the determinants of the level of OOP payments, the resulting financial risk and the probability of forgone healthcare in Bangladesh. This is the first attempt in Bangladesh to identify the potential factors which influence both financial burden and forgone healthcare using the most recent nationally representative household survey data. The findings suggested that 1 in 4 Bangladeshis incurred financial catastrophe and 14% population had forgone healthcare for any reason. Individuals having chronic illness and having higher economic status are the common determinants of OOP payments, CHE and forgone healthcare. Inpatient and outpatient public and private health services were key predictors for OOP payments and CHE.

Our study indicated that more than 1 in 10 Bangladeshis reported foregone healthcare in the last 12 months. Among those who do not seek care, the main reasons for foregone healthcare were related to treatment cost, permission, none to accompany, and distance to health facilities. Our study indicated that around 25% of households in Bangladesh incurred financial catastrophe when they received health services. Wealthy household, household member with chronic disease, both public and private health service users faced increased risk of catastrophic health payments. These findings are consistent with a previous study conducted in Bangladesh [13, 14, 25, 34–36]. Although the Bangladeshi public health facilities are highly subsidized, they fail to protect households against catastrophic health payments. In our study, household consumption quintile had a linear negative association with the forgoing of medical care for high treatment costs reasons, which is in consensus with results from previous studies [37]. Our study findings highlight that persons with lower income group in Bangladesh were at greater risk of forgoing treatment due to cost more frequently. Consistent with previous studies [38], elderly and people with chronic diseases were found to be more likely to forgo care. Given the sharply rising prevalence of chronic diseases in LMICs [20, 25, 39, 40], the expenditures for these conditions will add to the current burden of costs associated with infectious diseases, creating further challenges for households to obtain necessary care. Case in point, 17% of foregone care was attributed to affordability of medical fees or medications which raises concerns regarding the ability of Bangladesh health care system to cater for the low-income groups. This compared to only 3% of foregone care being associated with availability of health services such as distance further highlights the significance of direct healthcare payments as a barrier more than other dimensions of barriers in Bangladesh.

Being a developing country with majority rural residents, Bangladesh utilizes the concerted efforts of government, non-government, private and international organizations to deliver basic healthcare services for its population. However, despite such promising collaboration across the country, a high burden of OOP payment persists which demonstrates underlying myriad of contributing factors. To begin with, the poorly designed health financing system in Bangladesh continues to lack insurance policies which not only forces the general people to adopt distress financing for healthcare but also discourages them from seeking services when in need. The excessive financial burden often leads the families to modify their health service utilization pattern, and those who suffer from multiple chronic diseases consequently face worsening health conditions requiring vastly expensive treatments. Studies have shown that health insurance schemes not only successfully promote effective service utilization but also ensure simultaneous reduction in such relinquishment of care [41, 42]. The Government of Bangladesh has instead subsidized its public health facilities to a bare minimum cost of care, but unfortunately the hidden expenditures in forms of unofficial medical charges, financial incentives or tips to the porters and female helpers (ayas), and travel and food costs make institutional care an expensive experience for underserved populations [43]. Moreover, the frequent understaffing at the government health centers (due to absent healthcare providers at the sanctioned positions), hostile behavior of their available workforce, long waiting periods and inconvenient opening and closing times further restrain the poor people from using their services [44]. Concurrently, most impoverished health users suffer from indecisions, reduced perception of medical need, lack of awareness regarding the proper health center, financial uncertainty and perceived economic inadequacy – all of which cumulatively cause them to forgo essential healthcare and subsequently face devastating out-of-pocket expenditures.

This is the first study providing evidence of prevalence, inequalities and determinants of burden of OOP payments and foregone healthcare in the Bangladeshi population using recent large-scale nationwide survey data. Notwithstanding these strengths, is important to recognize the limitations of this study. First, the inability to estimate causal effects derives from the lack of control for reverse causality between predictors and occurrence of burden of OOP payments and forgone healthcare. Secondly, there may have been an issue of recall bias when respondents were asked if they limited medicines over a 12-month period.

## Conclusion and policy recommendations

Around one in four Bangladeshi incur catastrophic health expenditure, 14% population forgone healthcare, and treatment is the leading reason for forgone healthcare in Bangladesh. This study demonstrated that individuals who are chronically ill, users of either public or private health services, and belonging to wealthy socioeconomic condition have increased risks for OOP payment and catastrophic health expenditure. Forgone healthcare is also influenced by individual’s chronic illness, economic status, and place of residence. Socioeconomically disadvantaged population are reported more likely to forgo health services for high treatment costs in Bangladesh.

In Bangladesh’s current health financing system, the costs associated with chronic disease fall most severely upon those least able to afford them, increasing the risk of financial hardship and impoverishment for the families concerned. Advancement towards achieving national health goals will only be accelerated by:

- Ensuring affordability of health services, reducing geographical barriers, and improving acceptability, will be critical in reducing forgone healthcare.
- Increasing government spending on health, properly monitoring subsidized programs, and committing to health insurance for the whole population, for salaried workers in both public and private sectors, and voluntary memberships for dependents, farmers and self-employed persons, similarly to programs in Vietnam, and other developing countries
- Expanding benefits packages for poor and chronically ill people to include highly-subsidized or free hospital services.

## Supplementary Information

**Additional file 1: **Supplemental appendix. Table S1. Incidence of catastrophic health expenditure by different definitions and thresholds, Bangladesh (*n*=39,124). Table S2: Heckman regression results for the total amount of OOP health payments, Bangladesh 2017 (*N*=39,124).

## Data Availability

The is not publicly available. The corresponding author is fully responsible for data gathering.

## Abbreviations

CEA: Census enumerations area
CHE: Catastrophic health expenditure
CI: Confidence interval
HIES: Household Income and Expenditure Survey
LMICs: Low- and middle-income countries
OOP: Out-of-pocket payment
PSU: Primary sampling units
RR: Relative risk
SII: Slope index of inequality
