# Supplementary material for: Forgone healthcare and financial burden due to out-of-pocket payments in Bangladesh: a multilevel analysis
Source: Health Econ Rev. 2022 Jan 10;12:5. doi: 10.1186/s13561-021-00348-6 (PMC8751265; doi:10.1186/s13561-021-00348-6)
Supplement: Supplementary file 1 — Additional file 1: Supplemental appendix. Table S1. Incidence of catastrophic health expenditure by different definitions and thresholds, Bangladesh (n=39,124). Table S2: Heckman regression results for the total amount of OOP health payments, Bangladesh 2017 (N=39,124). [file 13561_2021_348_MOESM1_ESM.docx]

Supplemental appendix

Table S1: Incidence of catastrophic health expenditure by different definitions and thresholds, Bangladesh (n=39,124)

| Definition and cut-point | Frequency of catastrophic expenditure | Incidence of catastrophic expenditure  (95% confidence interval) |
| --- | --- | --- |
| Total consumption |  |  |
| >10 | 13,445 | 34.07 (32.66-35.51) |
| >15 | 9,053 | 23.21 (21.91-24.55) |
| Non-food consumption |  |  |
| >25 | 14,019 | 35.48 (34.01-36.98) |
| >40 | 9,556 | 24.57 (23.27-25.93) |
| Capacity to pay |  |  |
| >=40 | 5,008 | 12.24 (11.4-13.13) |

Table S2: Heckman regression results for the total amount of OOP health payments, Bangladesh 2017 (N=39,124)

| Variables | 1st stage | | |  | 2nd stage | | |
| --- | --- | --- | --- | --- | --- | --- | --- |
|  | Participation (probit) equation | | |  | Expenditure (regression) equation | | |
|  | Coefficient | SE | p-value |  | Coefficient | SE | p-value |
| Constant | 1.39 | 0.10 | <0.01 |  | 7.80 | 0.29 | <0.01 |
| Age, years |  |  |  |  |  |  |  |
| 0-4 | 0.00 | NA |  |  | 0.00 | NA |  |
| 5-9 | -0.03 | 0.07 | 0.69 |  | 0.01 | 0.05 | 0.80 |
| 10-14 | -0.28 | 0.07 | <0.01 |  | -0.11 | 0.05 | 0.03 |
| 15-19 | -0.32 | 0.08 | <0.01 |  | -0.07 | 0.06 | 0.24 |
| 20-24 | -0.59 | 0.08 | <0.01 |  | 0.00 | 0.08 | 0.99 |
| 25-64 | -0.47 | 0.09 | <0.01 |  | 0.00 | 0.07 | 0.99 |
| >=65 | -0.46 | 0.11 | <0.01 |  | 0.11 | 0.08 | 0.19 |
| Gender |  |  |  |  |  |  |  |
| Male | 0.00 | NA |  |  | 0.00 | NA |  |
| Female | 0.01 | 0.03 | 0.67 |  | 0.05 | 0.03 | 0.06 |
| Religion |  |  |  |  |  |  |  |
| Muslim | 0.00 | NA |  |  | 0.00 | NA |  |
| Non-Muslim | -0.25 | 0.14 | 0.07 |  | -0.05 | 0.05 | 0.33 |
| Marital status |  |  |  |  |  |  |  |
| Never married | 0.00 | NA |  |  | 0.00 | NA |  |
| Currently married | 0.24 | 0.08 | <0.01 |  | 0.07 | 0.06 | 0.18 |
| Widowed^a^ | 0.21 | 0.10 | 0.04 |  | -0.06 | 0.08 | 0.48 |
| Has a chronic disease |  |  |  |  |  |  |  |
| No | 0.00 | NA |  |  | 0.00 | NA |  |
| Yes | -0.08 | 0.06 | 0.17 |  | 0.15 | 0.03 | <0.01 |
| **Inpatient care** |  |  |  |  |  |  |  |
| None |  |  |  |  | 0.00 | NA |  |
| Public hospital/clinic |  |  |  |  | 0.71 | 0.08 | <0.01 |
| Private providers |  |  |  |  | 1.03 | 0.06 | <0.01 |
| **Outpatient care** | |  |  |  |  |  |  |
| None | |  |  |  | 0.00 | NA |  |
| Public hospital/clinic | |  |  |  | 0.86 | 0.06 | <0.01 |
| Private hospital/clinic | |  |  |  | 0.88 | 0.05 | <0.01 |
| Self-medication^b^ | |  |  |  | 0.26 | 0.05 | <0.01 |
| **Income earner** | |  |  |  |  |  |  |
| Yes | |  |  |  | 0.00 | NA |  |
| No |  |  |  |  | -0.04 | 0.03 | 0.17 |
| **Consumption quintile** |  |  |  |  |  |  |  |
| Q1 (poorest) | 0.00 | NA |  |  | 0.00 | NA |  |
| Q2 | 0.21 | 0.05 | <0.01 |  | 0.40 | 0.04 | <0.01 |
| Q3 | 0.29 | 0.06 | <0.01 |  | 0.70 | 0.04 | <0.01 |
| Q4 | 0.13 | 0.09 | 0.12 |  | 1.11 | 0.05 | <0.01 |
| Q5 (richest) | 0.00 | 0.09 | 0.98 |  | 1.84 | 0.05 | <0.01 |
| Household size | -0.03 | 0.01 | 0.06 |  | 0.13 | 0.01 | <0.01 |
| **Place of residence** |  |  |  |  |  |  |  |
| Rural | 0.00 | NA |  |  | 0.00 | NA |  |
| Urban | -0.18 | 0.09 | 0.04 |  | -0.20 | 0.04 | <0.01 |
| Mills ratio |  |  |  |  |  |  |  |
| rho | 0.018 | 0.011 |  |  |  |  |  |
| sigma | 1.196 | 0.014 |  |  |  |  |  |
| lambda | 0.022 | 0.014 |  |  |  |  |  |

^a^Widowed/divorced/separated; *Self-medication/pharmacy/traditional healer; SE, standard error
